# Short-Term Clinical Outcomes of Platelet-Rich Plasma Injections in Patients With Rotator Cuff Tendinopathy and Partial-Thickness Rotator Cuff Tears

**DOI:** 10.7759/cureus.105855

**Published:** 2026-03-25

**Authors:** Yoshiaki Nagumo, Hajime Inoue

**Affiliations:** 1 Orthopaedic Surgery and Regenerative Medicine, NAG Orthopaedic Clinic, Tokyo, JPN; 2 Center of Regenerative Medicine, Ginza Yoshie Clinic, Tokyo, JPN; 3 Division of Regenerative Medicine, Suiu Clinic Tokyo, Tokyo, JPN

**Keywords:** joints, non-surgical shoulder treatment, platelet rich plasma, prp therapy, regenerative medicine therapies, regenerative orthopedics, rotator cuff tears, rotator cuff tendon, shoulder injuries

## Abstract

Introduction: Platelet-rich plasma (PRP) has been increasingly applied for rotator cuff pathology; however, clinical evidence regarding its short-term efficacy remains limited. This study evaluated the effectiveness of a single PRP injection for rotator cuff injury.

Methods: This single-center retrospective study analyzed 25 cases managed between August 2024 and August 2025 to evaluate the effectiveness of a single PRP injection for rotator cuff injury. Pain was assessed using the visual analog scale (VAS), and shoulder function was assessed using Shoulder 36 (Version 1.3) at baseline and at one, two, and three months.

Results: Mean VAS decreased from 7.48 at baseline to 4.76 at one month, 4.40 at two months, and 3.16 at three months, with significant improvements at all follow-up time points compared with baseline (paired t-test, p < 0.001). At three months, 20 of 25 cases (80.0%) achieved a VAS improvement of ≥2 points, and 16 of 25 cases (64.0%) achieved a ≥50% reduction from baseline. In the Shoulder 36, the domains of pain, range of motion, muscle strength, general health perception, and activities of daily living improved over time; pain and muscle strength improved significantly from one month, and range of motion, general health perception, and activities of daily living improved significantly at two and three months (all p < 0.05), whereas the sports ability domain did not reach statistical significance. No significant associations were observed between three-month improvements in VAS or Shoulder 36 and the interval from symptom onset to the first PRP injection, baseline Ultrasound Shoulder Pathology Rating Scale (USPRS) grade, or prior treatment (no treatment, hyaluronic acid, or corticosteroid). Retreatment was performed in six cases (24.0%), and PRP was selected in all cases; baseline characteristics and three-month VAS improvement did not differ significantly between cases with and without retreatment.

Conclusion: In this single-center retrospective cohort of 25 cases, a single PRP injection was associated with significant improvements in VAS and multiple Shoulder36 domains over three months, and no apparent complications were observed during follow-up.

## Introduction

Shoulder pain is one of the most frequently encountered symptoms in clinical practice. Rotator cuff pathology is recognized as a major cause of shoulder pain and functional impairment [1], with a reported prevalence ranging from 13% to 32% [2].

Rotator cuff syndrome is a progressive condition, and it has been reported to carry the risk of progression not only to tear enlargement but also to tendon degeneration, fatty infiltration of the rotator cuff muscles, localized osteopenia of the proximal humerus, and degenerative changes of the glenohumeral joint [3]. In this context, treatment strategies aimed at achieving more rapid healing have been sought.

Conventional first-line management consists of physical therapy and nonsteroidal anti-inflammatory drugs (NSAIDs); in cases refractory to these treatments, corticosteroid injection into the subacromial space is considered [4]. Corticosteroids may facilitate the healing process in tendon disorders during the acute inflammatory phase; however, they have been reported to be associated with an increased risk of tendon rupture and suppression of collagen synthesis [5]. Accordingly, careful consideration is required when applying corticosteroid therapy in patients with rotator cuff injuries.

In recent years, platelet-rich plasma (PRP) has attracted increasing attention as a therapeutic modality containing a variety of biological factors capable of modulating tissue repair processes, and its clinical relevance has been actively investigated [6].

The present study was conducted to evaluate the efficacy of PRP therapy in the treatment of rotator cuff tears.

## Materials and methods

This study was a single-center retrospective observational study, analyzing data from 25 patients collected between August 2024 and August 2025. Among patients who presented to NAG Orthopaedic Clinic, Tokyo, Japan, with peri-shoulder pain during the observation period, those in whom the shoulder joint was strongly suspected as the primary pain source based on physical examination, who were subsequently diagnosed with rotator cuff injury by ultrasonography, and who had undergone PRP injection therapy with at least three months of post-treatment follow-up were enrolled in the study.

To evaluate the isolated therapeutic effect of PRP under standardized conditions, only patients who received a single PRP injection were included in this study. Multiple injections may act as a treatment-intensifying factor and introduce heterogeneity in treatment exposure, thereby confounding the interpretation of outcomes. Therefore, by restricting the analysis to single-injection cases, we aimed to improve internal validity and more accurately assess the effect of a single PRP intervention as an initial treatment.

The exclusion criteria (Table 1) were as follows: fractures or bone-related disorders, other definitive causes of shoulder pain such as gouty arthritis or rheumatoid arthritis, systemic diseases including hepatitis and hematologic disorders, active malignancy, pregnancy, suspected referred pain originating from cervical spine pathology, prior PRP therapy or stem cell joint injection to the shoulder, bilateral involvement (due to potential inaccuracy of questionnaire-based assessments), and cases with less than three months of follow-up.

**Table 1:** Exclusion criteria for patient selection.

Category	Exclusion criteria
Other causes of shoulder pain	Patients with other clear causes of shoulder pain, including fractures, bone disorders, gouty arthritis, or rheumatoid arthritis
Systemic diseases	Patients with systemic diseases such as hepatitis or hematologic disorders
Malignancy	Patients with active malignant disease
Pregnancy	Pregnant patients
Cervical spine–related pain	Patients with suspected referred pain originating from cervical spine pathology
Prior regenerative therapy	Patients who had previously received PRP therapy or stem cell intra-articular injections to the shoulder
Laterality	Patients with bilateral involvement (because accurate questionnaire-based evaluation could not be ensured)
Treatment interval	Patients with less than 3 months since the final PRP injection
Follow-up	Patients for whom follow-up of at least 3 months could not be obtained

Study design

The primary outcome measure was pain, assessed using the visual analog scale (VAS) [7,8]. Shoulder function was evaluated using the patient-reported outcome questionnaire Shoulder 36 (Version 1.3) [9,10], developed in 2010 by the Japanese Orthopaedic Association and the Japan Shoulder Society, and its use for study or research purposes is permitted free of charge with proper citation and version number; unauthorized modification is prohibited and commercial use requires permission from the Japanese Orthopaedic Association [9]. Shoulder 36 consists of 36 items across six domains (pain, range of motion, muscle strength, general health, activities of daily living, and sports ability). Each item is scored from 0 to 4 (higher scores indicate better status), and each domain score is calculated as the mean of the item scores within that domain; blank/invalid responses are excluded from the mean, but domains with fewer than half of the required responses are treated as invalid. Specific rules apply to the pain domain when fewer than half of the responses are available.

Shoulder ultrasonography was performed using a high-frequency L64 Linear Transducer (18-5 MHz) (Fujifilm Holdings Corporation, Tokyo, Japan). Patients were examined in the seated position, and both transverse and longitudinal scans were obtained to assess the rotator cuff tendons. All ultrasonographic image evaluations in this study were conducted by a single author, with no involvement of other physicians. Ultrasonographic findings of the rotator cuff were classified by partially adopting the previously reported Ultrasound Shoulder Pathology Rating Scale (USPRS) [11]. Supraspinatus tendon pathology was graded on a scale from 0 to 6 as follows: Grade 0, normal; Grade 1, mild tendinopathy; Grade 2, severe tendinopathy; Grade 3, intratendinous abnormality; Grade 4, partial-thickness tear; Grade 5, focal full-thickness tear involving both the bursal and articular sides with reduced tendon volume; and Grade 6, massive full-thickness tear with tendon nonvisualization and retraction.

PRP technique

PRP was prepared using a PRP preparation kit (Condensia®, KYOCERA Medical Corporation, Kyoto, Japan). Under sterile conditions, venous blood was collected from each patient, and 18 mL of blood was mixed with 2 mL of citrate dextrose anticoagulant. The mixture was subjected to a double-spin centrifugation process (first centrifugation: 600 × g for seven minutes, second centrifugation: 2,000 × g for five minutes) to obtain a final volume of 1.5 mL of leukocyte-poor PRP. In this study, quantitative measurements of platelet concentration and leukocyte count were not performed; however, previous reports on this system have demonstrated an approximately sevenfold increase in platelet concentration relative to whole blood, with low leukocyte content [12]. The resulting PRP was injected into the glenohumeral joint under ultrasound guidance. The injection procedure was conducted based on previously published studies [13-15].

After the injection, patients were instructed to refrain from rotational movements of the shoulder for one week. Immediate icing of the treated area was performed in the clinic, and no analgesic medications were prescribed. During the observation period, no physiotherapy was provided, and except for the aforementioned activity restriction period, patients were allowed to perform daily activities and exercise without limitation within their pain tolerance. However, high-load pressing movements were prohibited for one month after the final PRP injection. Patients were followed up at four, eight, and 12 weeks after PRP administration. Outcome assessments included the VAS and Shoulder 36 (Version 1.3).

Statistical analysis

All data were reviewed and then statistically analyzed using Python (pandas and SciPy libraries) (Python Software Foundation, Wilmington, Delaware, United States). Descriptive statistics were calculated for all variables; categorical variables are presented as counts and percentages. Continuous variables are presented as ranges, means, and standard deviations (SD). Comparisons across time points (baseline, one month, two months, and three months) were treated as paired data. Normality of the within-subject differences was assessed using the Shapiro-Wilk test; paired t-tests were used for normally distributed differences, and the Wilcoxon signed-rank test was used for non-normally distributed differences. Associations between variables were evaluated using Spearman’s rank correlation coefficient. A p-value < 0.05 was considered statistically significant. Figures and tables were created using matplotlib. (https://matplotlib.org/).

## Results

A total of 25 participants were included in the study, comprising 14 men and 11 women. The age ranged from 29 to 74 years, with a mean age of 52.9 ± 12.2 years. Eighteen cases involved the right shoulder and seven involved the left shoulder. Pre-PRP treatments included no prior treatment in 13 cases (52.0%), hyaluronic acid injections in six cases (24.0%), and corticosteroid injections in six cases (24.0%). The interval from symptom onset to the first PRP injection ranged from 0.5 to 96 months, with a median of three months (Table 2).

**Table 2:** Baseline demographic and clinical characteristics of the study participants (N = 25).

Variable	Category	Values
Sex, n (%)	Male	14 (56%)
	Female	11 (44%)
Age (years)	Range	29-74
	Mean ± SD	52.9±12.2
Laterality, n (%)	Right	18 (72%)
	Left	7 (28%)
Pre-PRP treatment, n (%)	None	13 (52%)
	Hyaluronic acid	6 (24%)
	Steroid	6 (24%)
Symptom duration (months)	Range	0.5-96
	Median	3

Regarding comorbidities, only the presence of malignancy was used as an exclusion criterion, and other medical histories were recorded but not included in the analysis (Table 1). Baseline USPRS grades were Grade 2 (severe tendinopathy) in five cases (20.0%), Grade 3 (intratendinous abnormality) in four cases (16.0%), and Grade 4 (partial tear) in 16 cases (64.0%) (Table 3).

**Table 3:** Distribution of pre-injection USPRS grades (N=25). USPRS: Ultrasound Shoulder Pathology Rating Scale

USPRS grade	Frequency	Percentage
Grade 2 (Severe tendinosis)	5	20
Grade 3 (Intrasubstance abnormality)	4	16
Grade 4 (Partial tear)	16	64
Total	25	100

**Figure 1:**
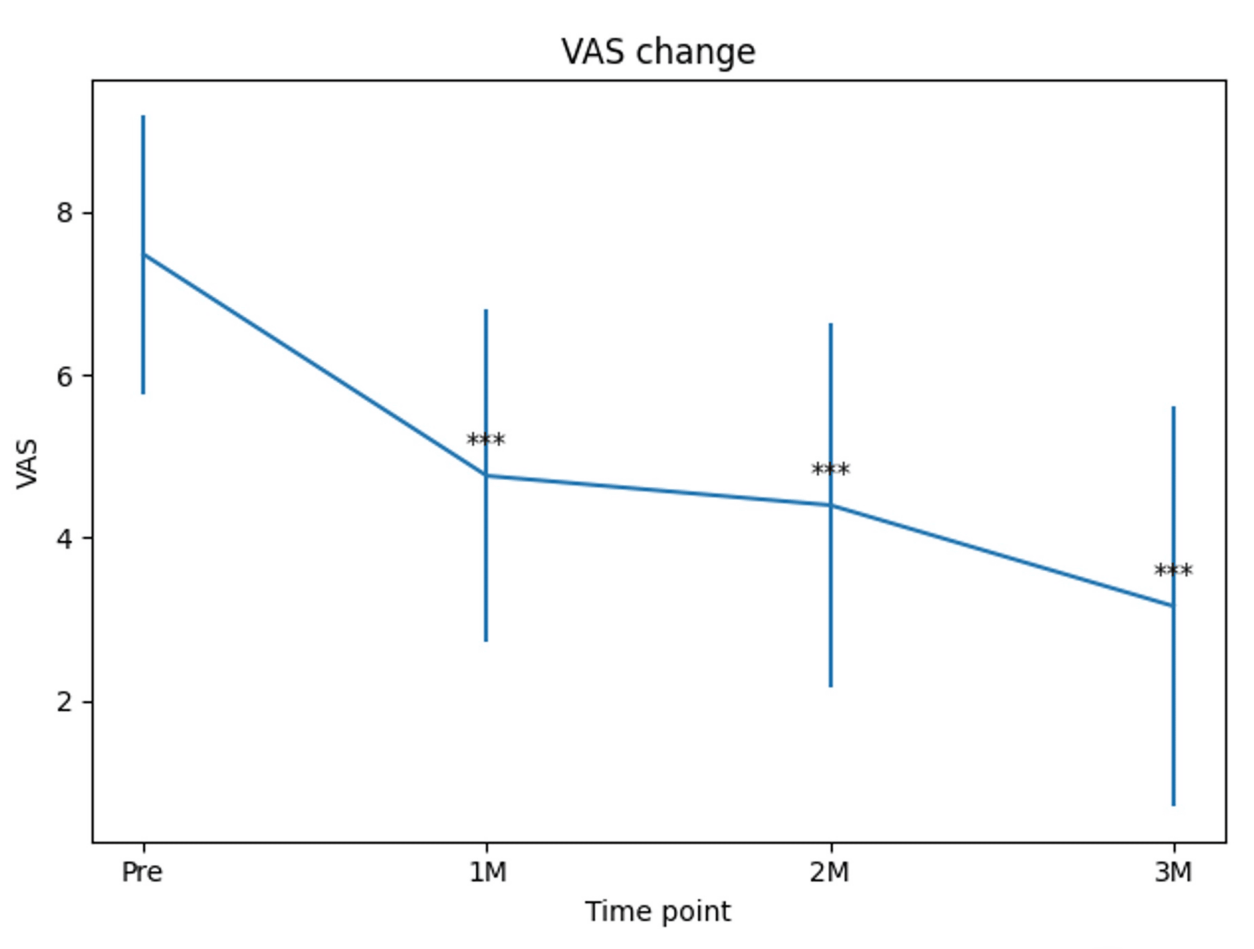
Changes in VAS scores after PRP injection. Error bars represent standard deviations. Statistical comparisons were performed between baseline and each follow-up time point using paired t-tests, and all post-treatment scores showed significant improvement compared with baseline (*p < 0.001). VAS: visual analog scale; PRP: platelet-rich plasma

The mean VAS score was 7.48 at baseline. It decreased significantly to 4.76 at one month, 4.40 at two months, and 3.16 at three months. Paired t-tests demonstrated statistically significant improvements from baseline to one month (difference 2.72 ± 2.54, p = 0.000017), two months (difference 3.08 ± 2.60, p = 0.000004), and three months (difference 4.32 ± 2.81, p = 0.000000063) (Figure 1). At three months, 20 cases (80.0%) showed a VAS improvement of at least 2 points, and 16 cases (64.0%) demonstrated a reduction of 50% or more compared with baseline. The minimal clinically important difference (MCID) for VAS pain has been reported to be approximately 1.4 points [16], and the mean improvement observed in this study (4.32 points) exceeded this threshold.

**Figure 2:**
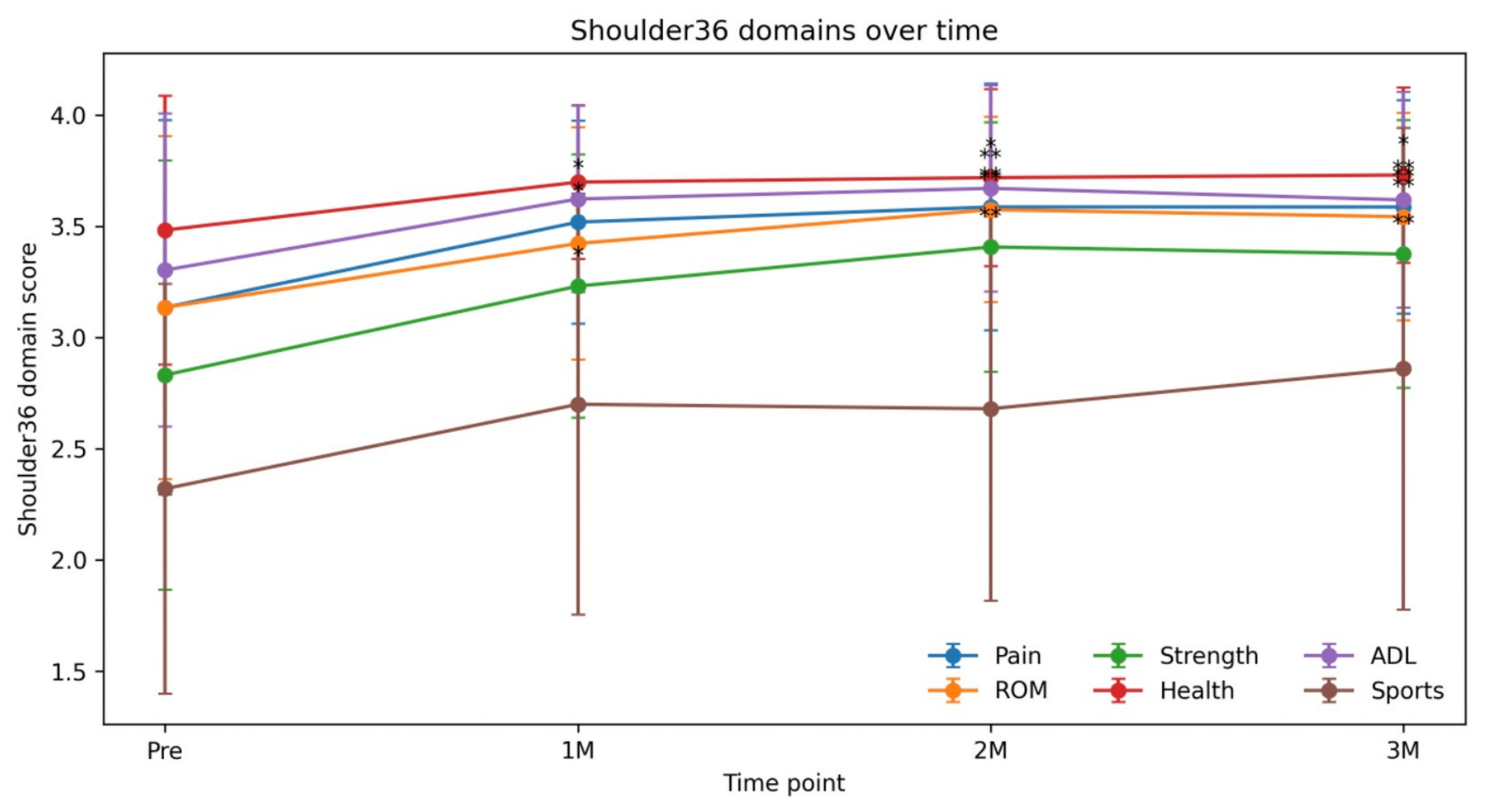
Time-course changes in Shoulder 36 (Version 1.3) domain scores after PRP injection. The domains of pain, range of motion, muscle strength, general health perception, and activities of daily living showed stepwise improvement over the three-month follow-up period compared with baseline. Pain and muscle strength demonstrated significant improvement from one month, while range of motion, general health perception, and activities of daily living showed significant improvement at two and three months. In contrast, although sports ability exhibited a trend toward improvement, it did not reach statistical significance. Error bars represent standard deviations. Use of Shoulder 36 (Version 1.3) for for study or research purposes is permitted free of charge with proper citation and version number; unauthorized modification is prohibited and commercial use requires permission from the Japanese Orthopaedic Association [9,10]. Asterisks indicate statistically significant differences compared with baseline (*p < 0.05, **p < 0.01, ***p < 0.001, paired t-test). PRP: platelet-rich plasma; ROM: range of motion; ADL: activities of daily living

All domains of the Shoulder 36, including pain, range of motion, muscle strength, general health perception, and activities of daily living, showed progressive improvement over time compared with baseline. Pain and muscle strength improved significantly from one month, while range of motion, general health perception, and activities of daily living showed significant improvement at two and three months (all p < 0.05). Detailed domain scores are summarized in Table 4, and time-course changes are illustrated in Figure 2. In contrast, the sports ability domain did not show statistically significant differences at any evaluation time point. No established MCID has been reported for the Shoulder 36.

**Table 4:** Time-course changes in Shoulder 36 (Version 1.3) domain scores at baseine and at one, two, and three months after PRP injection. Pain and muscle strength showed significant improvement compared with baseline from one month onward, while range of motion, general health perception, and activities of daily living demonstrated significant improvement at two and three months. In contrast, although sports ability showed a trend toward improvement, no statistically significant differences were observed at any evaluation time point. Use of Shoulder 36 (Version 1.3) for research is permitted free of charge [9,10]. Asterisk (*) indicates a statistically significant difference compared with baseline (p < 0.05, paired t-test). PRP: platelet-rich plasma

Domain	Baseline (Mean ± SD)	1 month	2 months	3 months
Pain	3.14 ± 0.84	3.52 ± 0.46*	3.59 ± 0.55*	3.59 ± 0.48*
Range of motion	3.14 ± 0.77	3.42 ± 0.52	3.58 ± 0.42*	3.54 ± 0.47*
Strength	2.83 ± 0.97	3.23 ± 0.59*	3.41 ± 0.56*	3.38 ± 0.60*
General health	3.48 ± 0.61	3.70 ± 0.35	3.72 ± 0.40*	3.73 ± 0.39*
Activities of daily living	3.30 ± 0.70	3.65 ± 0.41*	3.71 ± 0.43*	3.67 ± 0.42*
Sports	2.32 ± 0.92	2.70 ± 0.95	2.68 ± 0.86	2.86 ± 1.09

No significant association was observed between the interval from symptom onset to the first PRP injection (months) and the VAS improvement at three months (VAS_pre − VAS_3M) (Spearman’s rank correlation coefficient ρ = 0.007, p = 0.972). The three-month VAS improvement stratified by baseline USPRS grade (Grade 2/3/4) was 2.50 ± 1.91 (n = 4) for Grade 2, 5.60 ± 2.30 (n = 5) for Grade 3, and 4.38 ± 3.01 (n = 16) for Grade 4, with no significant differences among groups (Kruskal-Wallis test, p = 0.227). Similarly, when stratified by prior treatment before PRP (no treatment/hyaluronic acid/corticosteroid), the 3-month VAS improvement was 4.47 ± 2.90 (n = 15) in the no-treatment group, 1.00 ± 1.73 (n = 3) in the hyaluronic acid group, and 5.43 ± 1.99 (n = 7) in the corticosteroid group, with no significant intergroup differences (Kruskal-Wallis test, p = 0.116).

Likewise, no significant association was found between the interval from symptom onset to the first PRP injection (months) and the Shoulder36 improvement at three months (S36_3M − S36_pre) (Spearman’s rank correlation coefficient ρ = 0.144, p = 0.494). The Shoulder36 improvement stratified by baseline USPRS grade was 0.269 ± 0.660 (n = 4) for Grade 2, 0.613 ± 0.893 (n = 5) for Grade 3, and 0.404 ± 0.549 (n = 16), with no significant differences among groups (Kruskal-Wallis test, p = 0.579). When stratified by prior treatment before PRP, the Shoulder36 improvement was 0.517 ± 0.760 (n = 15) in the no-treatment group, 0.161 ± 0.113 (n = 3) in the hyaluronic acid group, and 0.340 ± 0.365 (n = 7) in the corticosteroid group, with no significant intergroup differences (Kruskal-Wallis test, p = 0.550).

No apparent complications were observed throughout the follow-up period, and no cases showed symptom exacerbation after treatment. Retreatment was performed in six cases (24.0%), and PRP was selected in all cases. Comparisons of baseline characteristics according to retreatment status revealed no significant differences in age (median: 52 years in the no-retreatment group vs. 57 years in the retreatment group, p = 0.503), symptom duration (median: 3.0 months vs. 1.5 months, p = 0.156), baseline VAS (median: 8.0 vs. 7.5, p = 0.278), or three-month VAS improvement (median: 5.0 vs. 3.5, p = 0.630) (all Mann-Whitney U tests).

## Discussion

When conventional intra-articular treatments with hyaluronic acid or corticosteroid injections are ineffective, or when patients do not wish to undergo these therapies, autologous tissue-based joint treatments are considered as alternative options. Among these, PRP therapy has become widely adopted because the preparation process is relatively simple and the financial burden is comparatively low.

Several studies have previously investigated the analgesic effects of PRP in patients with rotator cuff injuries. In a double-blind randomized controlled trial conducted by Dadgostar et al., 58 patients with rotator cuff tendinitis/tendinopathy diagnosed by MRI and other imaging modalities were randomly assigned to a PRP group or a corticosteroid group [17]. Following a single injection into the subacromial space, VAS scores improved significantly in both groups; however, pain reduction at three months was greater in the PRP group. In a prospective cohort study by Prodromos et al. involving 71 shoulders with rotator cuff injuries refractory to conservative treatment, dual PRP injections were administered into the supraspinatus tendon insertion and the glenohumeral joint, and clinical outcomes were evaluated, and Global Rating, VAS, and QuickDASH (Quick Disabilities of the Arm, Shoulder, and Hand) scores all showed significant improvement at six, 12, and 24 months compared with baseline [18]. Furthermore, treatment effects were greater in cases with more severe structural damage, with the most marked improvement in Global Rating observed in the group with partial tears exceeding 50%. Although the present study was a single-arm interventional trial without a control group, pain reduction was observed in all cases, which is consistent with previous reports. In contrast, no significant association was found between baseline USPRS and the magnitude of pain improvement (VAS) or functional improvement (Shoulder 36) at three months. This may be attributable to the short-term nature of the three-month evaluation in the present study, compared with the mid- to long-term assessments including Global Rating at six, 12, and 24 months reported by Prodromos et al. [18], as well as the small sample sizes in USPRS Grade 2 (n = 4) and Grade 3 (n = 5) and the limited severity range restricted to Grades 2-4.

Among rotator cuff injuries, the supraspinatus tendon is known to be a site where tissue healing is particularly difficult due to the presence of a critical zone at the tendon insertion. Although large-scale studies clearly demonstrating significant structural repair of rotator cuff lesions by PRP have not been identified to date, there are reports at the animal experimental level evaluating the potential effects of PRP on rotator cuff healing. For example, in a rat rotator cuff repair model reported by Hapa et al., local administration of autologous PRP suppressed inflammation and angiogenesis, improved continuity of the tendon-bone interface at two weeks postoperatively, and increased mechanical strength [19]. These findings suggest that PRP may have promoted early healing remodeling at the tendon-bone insertion site.

In addition, in a study by El Gharbawy et al. involving a single PRP injection in 30 patients with rotator cuff injuries, a significant reduction in VAS scores was observed at four weeks (from 7.1 ± 0.99 before injection to 3.7 ± 1.09 after injection), along with highly statistically significant improvement in USPRS, which showed a significant positive correlation with the magnitude of VAS improvement [20]. In the present study, clinical outcomes after PRP administration were evaluated with a focus on pain and functional improvement, and structural regeneration or repair of the rotator cuff tissue, assessed by imaging, was not included as an outcome measure. Future studies incorporating imaging assessments are warranted to further investigate the effects of PRP therapy on the regenerative processes of rotator cuff tissue.

In the present study, no cases were observed in which symptoms clearly worsened, or PRP treatment was judged to be ineffective after therapy. However, a certain number of patients did not achieve clinically sufficient pain reduction or functional improvement. Because the interval from symptom onset to the first PRP injection, baseline USPRS grade, and prior treatment before PRP were not significantly associated with the magnitude of pain improvement (VAS) or functional improvement (Shoulder 36) at three months, it was not possible to identify factors associated with limited treatment response based solely on the variables analyzed in this study. In addition, no established indicators or guidelines currently exist to screen such cases in advance. Nevertheless, several clinical factors suggesting a lower likelihood of treatment efficacy have been reported in previous studies. For example, Saravanan et al. reported that the therapeutic effect of a single PRP treatment was inferior in patients aged 60 years or older, those with symptom duration longer than six months, or those with diabetes mellitus compared with patients who did not meet these conditions [21]. Prodromos et al. reported in their study of PRP therapy for patients with rotator cuff injuries that cases characterized primarily by inflammation without structural damage showed significantly less pain relief compared with those with structural lesions [10]. Although the underlying mechanism remains unclear, similar findings have been reported in the field of arthroplasty, where patients with milder preoperative radiographic changes have poorer postoperative outcomes; unlike severe osteoarthritis, mild osteoarthritis may involve pain amplification mediated by central sensitization in addition to organic structural changes [22]. Based on this concept, it is conceivable that, in the present study, cases with more pronounced underlying periarticular inflammatory changes may likewise have been less likely to achieve substantial analgesic effects, consistent with previous reports.

Meanwhile, retreatment was performed in six cases (24.0%), and PRP was selected in all cases based on patient preference. Comparisons of baseline characteristics according to the presence or absence of retreatment revealed no significant differences in age, symptom duration, baseline VAS, or the magnitude of VAS improvement at three months. Cases that underwent retreatment were not limited to those with pain that remained difficult to control at three months; a substantial number of patients had experienced pain reduction to a tolerable level but nevertheless evaluated the initial treatment effect positively and requested additional therapy in expectation of further symptom improvement. At our institution, the decision to perform retreatment was not based on pain or functional scores but was made in accordance with patient preference, and thus, the presence or absence of retreatment should not be interpreted as a direct indicator of treatment efficacy.

Reported sources of stem cells include dental pulp, bone marrow, adipose tissue, among others; however, for joint treatment in adults, adipose tissue is currently the most commonly used donor source from the perspectives of donor-site invasiveness and safety [23]. Therefore, unless otherwise specified in this manuscript, the term “stem cells” refers to adipose tissue-derived mesenchymal stem cells (ADSCs).

To the best of the authors’ knowledge, no studies have been reported comparing ADSCs and PRP as monotherapies for rotator cuff injuries; however, several studies have been published regarding the knee joint. In the study by Khoury et al., patients with knee osteoarthritis of Kellgren-Lawrence grade 1-3 received monthly intra-articular injections for a total of three sessions (ADSCs: approximately 43.8 × 10⁶ cells in total; PRP: 5.76-fold concentration) [24]. As a result, the PRP group showed significant deterioration in KOOS (Knee injury and Osteoarthritis Outcome Score) after six months, whereas the ADSC group maintained favorable outcomes up to 24 months. In addition, a systematic review and meta-analysis of six controlled clinical trials involving a total of 493 patients with knee osteoarthritis reported that combined therapy with mesenchymal stem cells (MSCs) and PRP resulted in significantly greater improvements in VAS at 6 and 12 months compared with MSC monotherapy and PRP monotherapy, and also demonstrated superior improvements in Western Ontario and McMaster Universities Osteoarthritis Index (WOMAC) scores at three and six months [25]. The therapeutic effects of MSCs are influenced by the intra-articular microenvironment, and their proliferation and differentiation are inhibited in highly inflammatory conditions; therefore, these findings may be interpreted as indicating that PRP improved the intra-articular environment and consequently promoted MSC proliferation and differentiation.

Thus, several treatment options may be considered when the effect of PRP is insufficient; however, no standardized therapeutic guidelines exist regarding the selection between PRP and ADSCs, and decisions are currently left to the clinical judgment of each institution and practitioner. In the present study, all six cases that proceeded to retreatment received prior explanations regarding both PRP and ADSC therapies, yet all patients elected to undergo repeat PRP treatment. Although the specific reasons for treatment selection were not systematically assessed, it is possible that time constraints related to cell culture and the cost associated with ADSC therapy influenced patient decision-making.

Limitations

The present study has several methodological limitations. The absence of a control group and randomization, as well as the lack of comparisons with corticosteroid injections or placebo, the small sample size of 25 cases, and the single-center, non-blinded design make it difficult to completely exclude the influence of assessment bias. In addition, because this was a single-arm observational study, the contribution of placebo effects or the natural course of symptom improvement to the observed improvements in pain and function cannot be completely excluded. Furthermore, the follow-up period was limited to three months, and thus, the long-term therapeutic effects and the potential for preventing progression to full-thickness tears remain unclear. Moreover, ultrasound evaluation is inherently operator-dependent and may be affected by interobserver variability and potential observer bias. Evaluation of imaging changes before and after PRP administration also remains an issue to be addressed in future studies. On the other hand, the study population consisted of cases refractory to conservative treatment, suggesting that the observed pain improvement is unlikely to be explained solely by the natural course and may be attributable to PRP injection.

## Conclusions

PRP injection demonstrated favorable safety and provided short-term pain relief in patients with rotator cuff tendinopathy and partial-thickness rotator cuff tears. This treatment may serve as an option that contributes to avoiding surgery through symptom improvement and may be considered as part of conservative management for patients with rotator cuff tendinopathy or partial-thickness rotator cuff tears.
